# Effect of Corilagin on the Proliferation and NF-*κ*B in U251 Glioblastoma Cells and U251 Glioblastoma Stem-Like Cells

**DOI:** 10.1155/2016/1418309

**Published:** 2016-05-09

**Authors:** Wen-Tao Yang, Gen-Hua Li, Zheng-You Li, Song Feng, Xue-Qin Liu, Guang-Kui Han, Hao Zhang, Xian-Yun Qin, Ran Zhang, Quan-Min Nie, Feng Jin

**Affiliations:** Department of Neurosurgery, Affiliated Hospital of Jining Medical University and Shangdong Provincial Key Laboratory of Stem Cells and Neuro-Oncology, Jining 272029, China

## Abstract

*Background*. This study is to explore the effect of corilagin on the proliferation and NF-*κ*B signaling pathway in U251 glioblastoma cells and U251 glioblastoma stem-like cells.* Methods*. CD133 positive U251 glioblastoma cells were separated by immunomagnetic beads to isolate glioblastoma stem-like cells. U251 cells and stem-like cells were intervened by different corilagin concentrations (0, 25, 50, and 100 *μ*g/mL) for 48 h, respectively. Cell morphology, cell counting kit-8 assay, flow cytometry, dual luciferase reporter assay, and a western blot were used to detect and analyze the cell proliferation and cell cycle and investigate the expression of IKB*α* protein in cytoplasm and NF-*κ*B/p65 in nucleus.* Results*. Corilagin inhibited the cell proliferation of U251 cells and their stem-like cells and the inhibition role was stronger in U251 stem-like cells (*P* < 0.05). The cell cycle was arrested at G2/M phase in the U251 cells following corilagin intervention; the proportion of cells in G2/M phase increased as the concentration of corilagin increased (*P* < 0.05). The U251 stem-like cells were arrested at the S phase following treatment with corilagin; the proportion of cells in the S phase increased as the concentration of corilagin increased (*P* < 0.05). The ratio of dual luciferase activities of U251 stem-like cells was lower than that of U251 cells in the same corilagin concentration. With increasing concentrations of corilagin, the IKB*α* expression in cytoplasm of U251 cells and U251 stem-like cells was increased, but the p65 expression in nucleus of U251 cells and U251 stem-like cells was decreased (*P* < 0.05).* Conclusion*. Corilagin can inhibit the proliferation of glioblastoma cells and glioblastoma stem-like cells; the inhibition on glioblastoma stem-like cell proliferation is stronger than glioblastoma cells. This different result indicates that the effect of corilagin on U251 cells and U251 stem-like cells may have close relationships with mechanism of cell cycle and NF-*κ*B signaling pathway; however, the real antitumor mechanism of corilagin is not yet clear and requires further study.

## 1. Introduction

Glioblastoma Multiforme (GBM) is the most devastating type of adult primary malignant brain tumors which is featured with high malignancy and poor prognosis, resulting in a median survival of 12–15 months [1]. More and more studies had shown that there were a small number of glioblastoma stem-like cells existing in glioblastoma cells. Glioblastoma stem-like cells have characteristics of self-renewal, unlimited proliferation, and multidirectional differentiation, which are considered the source of glioblastoma occurrence, development, recurrence, and drug resistance; the stem-like cells are becoming the new tool for the study and treatment of glioblastoma [2–4]. Corilagin is a member of polyphenolic tannins extracted from the Euphorbiaceae* Phyllanthus* plants. The molecular formula of corilagin is C_27_H_22_O_18_ [5]. Corilagin has strong antitumor [6], anti-inflammatory [7], antioxidation [8], thrombolytic and antihypertensive [9], hepatoprotective, and antiatherogenic [10] effects. In recent years, many literatures have reported that corilagin have significant antitumor effect on a variety of malignant tumor cells, but there is few research of corilagin on glioblastoma stem-like cells and glioblastoma stem-like cells.

## 2. Materials and Methods

### 2.1. Chemicals and Reagents

Corilagin standard substance (purity > 99%) for cells and PVDF membranes were purchased from Sigma (St. Louis, Mo, USA). Dulbecco's Modified Eagle's Medium/Nutrient Mixture F-12 Ham's (DMEM/F12) and fetal bovine serum (FBS) were purchased from HyClone (Logan, UT, USA). Trypsin as well as B-27 (50x) Serum-Free Supplement was purchased from Gibco (Grand Island, NY, USA). Epidermal growth factor (EGF), basic fibroblast growth factor (bFGF), and leukemia inhibitory factor (LIF) were purchased from PeproTech (Rocky Hill, NJ, USA). Rabbit anti-human nestin, rabbit anti-human glial fibrillary acidic protein (GFAP), mouse anti-human *β*-tubulin antibody, goat anti-mouse IgG-FITC antibody, and goat anti-rabbit IgG-FITC antibody were purchased from Wuhan Boster Biological Engineering Co., Ltd. (Wuhan, China). The primary mouse monoclonal antibodies to p65 subunit of nuclear factor kappa B (NF-*κ*B), IKB*α* (an endogenous inhibitor of NF-*κ*B), *β*-actin, and Histone H3 were purchased from Cell Signaling Technology (Beverly, MA, USA). The secondary antibody (an anti-mouse horse radish peroxidase-conjugated antibody) was purchased from Beijing Zhongshan Golden Bridge Biotechnology Co., Ltd. (Beijing, China). The CD133 cell isolation kit was purchased from Miltenyi Biotec GmbH (Bergisch Gladbach, Germany). The cell counting kit-8 (CCK-8), the nucleus and cytoplasm protein extraction kit, BCA protein assay kit, enhanced chemiluminescence kit, and cell cycle analysis kit were purchased from Shanghai Beyotime Biotechnology Institute (Shanghai, China). Dual luciferase reporter assay system was purchased from Promega (Beijing) Biotechnology Co., Ltd. (Beijing, China). The pRL-TK renilla luciferase reporter vector as well as pGL3-basic firefly luciferase reporter vector was designed by Shanghai Han Biotech Co., Ltd. (Shanghai, China).

### 2.2. Cell Culture Methods

#### 2.2.1. U251 Cell Line Culture

The cell line was cultured in a medium containing DMEM/F12 and 10% FBS, at 37°C in a humidified 5% CO_2_ atmosphere. The medium was renewed every 2-3 days.

#### 2.2.2. U251 Stem-Like Cell Sphere Culture

The U251 cells were inoculated into the medium (neural stem cell (NSC) medium) containing DMEM/F12, 20 ng/mL EGF, 20 ng/mL bFGF, 10 ng/mL LIF, and B-27 (1x), at 37°C in a humidified 5% CO_2_ atmosphere. Half of the medium was replaced once every 3-4 days.

### 2.3. Isolation of CD133+ U251 Stem-Like Cells

 Neurospheres were collected after they had grown in a large number of proliferation instances, digested by 0.25% trypsin for 2min then terminating digestion by 10% FBS. The cell suspension was filtered through a 200-mesh sieve and centrifuged at 1000 r.p.m. After disposing of supernatant, the primary glioblastoma cells were washed again with serum-free medium. Then the CD133+ cells were separated according to the instructions of the CD133 cell isolation kit. The CD133+ cells were collected and inoculated into NSC medium.

### 2.4. Differentiated U251 Stem-Like Cells Culture

The well-grown CD133+ cell spheres were selected and inoculated on 10% polylysine-coated slides and then were cultured in the medium containing DMEM/F12 and 10% FBS, at 37°C in a humidified 5% CO_2_ atmosphere.

### 2.5. Immunofluorescence Staining on CD133+ U251 Stem-Like Cell Spheres

The well-grown CD133+ cell spheres were selected and inoculated on 10% polylysine-coated slides. After drying at 37°C, the slides were washed by phosphate buffered saline (PBS) for 3 times. At room temperature, the cells were fixed by 4% paraform for 30 minutes and then washed by PBS again for 3 times. The rabbit anti-human nestin (1 : 200) (1st antibody) was added after being blocked by 5% goat serum at 37°C for 30 minutes, and the cells were placed at 4°C wet box all night. In the next following day the goat anti-rabbit IgG-FITC antibody (secondary antibody) was added for incubation for 30 minutes at 37°C after being washed by PBS for 3 times. At the same time, the PBS was used as negative control instead of 2nd antibody. The slides were washed by PBS again and then observed with an Olympus IX71 fluorescence microscope (Olympus, Tokyo, Japan). The procedure of immunofluorescence assay on GFAP as well as *β*-tubulin on differentiated U251 stem-like cells was as described for nestin except for respective antibodies.

### 2.6. Cell Morphology Observation

The U251 cells and U251 stem-like cells were inoculated into 6-well plates. Corilagin (100 *μ*g/mL) was added to the U251 cells and U251 stem-like cells for 48 h, and then the cell morphology was observed with an inverted microscope (Olympus IX71).

### 2.7. CCK-8 Assay

Cells (1 × 10^5^) were inoculated into 96-well plates; then corilagin (0, 25, 50, 100 *μ*g/mL) had been added to the U251 cells and U251 stem-like cells for 48 h. According to the instructions of CCK-8, cells were treated with 10 *μ*L CCK-8 solution and incubated for 2 h at 37°C. The absorbance (*A*) of each well was quantified at 450 nm using a microplate reader (Thermo Fisher Scientific, Shanghai, China). The cell survival rate (%) was calculated as follows: [*A*(experimental well) − *A*(blank well)]/[*A*(control well) − *A*(blank well)] × 100.

### 2.8. Cell Cycle Assay

The U251 cells and U251 stem-like cells were inoculated into 6-well plates and incubated with corilagin (0, 25, 50, and 100 *μ*g/mL) for 48 h. Then the cells were washed by ice-cold PBS and fixed with 70% (v/v) ice-cold ethanol overnight at 4°C. Next following day, the cells were detected by flow cytometry according to the instruction of cell cycle analysis kit. The cell cycle information was analyzed by ModFit LT 4.0 software.

### 2.9. Dual Luciferase Assay

The p65 gene promoter PCR product was cloned into pGL3-basic firefly luciferase reporter vector. The U251 cells and U251 stem-like cells (1 × 10^5^) for 24 h were inoculated into 24-well plates. In cells, a pRL-TK renilla luciferase reporter vector was cotransduced with the pGL3-basic vector for 24 h. We added corilagin (0, 25, 50, and 100 *μ*g/mL) to cells for 48 h and then harvested the cells for subsequent analysis. The dual luciferase assays were performed according to the instructions of dual luciferase reporter assay system. All transfection assays were performed in triplicate. For each cotransduction of pGL3-basic vector, the relative luciferase activities were normalized to the luciferase activity of pRL-TK.

### 2.10. Western Blotting Assay

Corilagin (0, 25, 50, and 100 *μ*g/mL) was added to the U251 cells and U251 stem-like cells which were inoculated in 6-well plates. 48 h later, the nucleus and cytoplasm protein were extracted according to the instruction of nucleus and cytoplasm protein extraction kit after cells were harvested and washed twice in PBS. Protein concentration was determined using BCA protein assay kit.

After separating in 10% SDS-PAGE gel, the nucleus and cytoplasm proteins were transferred to PVDF membranes. Then the membranes were incubated with anti-p65, anti-IKB*α*, anti-*β*-actin, or anti-Histone H3 for whole night at 4°C after they were blocked with 5% nonfat dried milk at room temperature for 1 h. Next following day, membranes were washed three times with TBST (10 mM Tris, pH 8.0, 150 mM NaCl, and 0.1% Tween 20) and incubated with anti-mouse horse radish peroxidase-conjugated antibody for 1 h at room temperature. The blot was detected by enhanced chemiluminescence kit according to manufacturer's instruction.

### 2.11. Statistical Analysis

Each experiment was repeated in triplicate. All data were expressed as mean ± standard deviation (SD). Statistical analysis was performed using SPSS 13.0 and comparisons of the data were performed with Student *t*-test. The results were presented as mean ± SD. *P* < 0.05 was considered to be statistical significance.

## 3. Results

### 3.1. Cell Morphology

**Figure 1:**
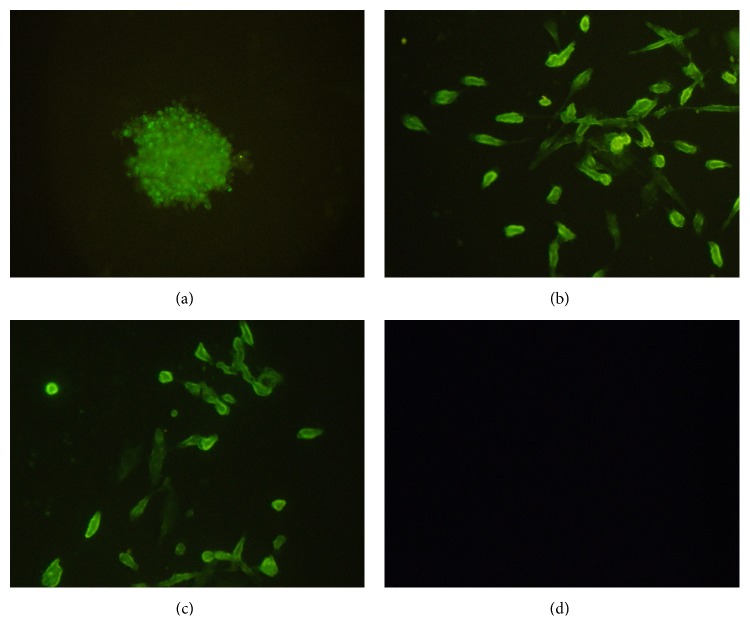
Immunofluorescence staining on CD133+ U251 stem-like cell spheres and the differentiation cells (10 × 20). (a) The stem-like cell spheres were stained positive for nestin. The differentiation cells stained positive for (b) GFAP and (c) *β*-tubulin. (d) Negative control cells.

CD133+ stem-like cells which were separated from U251 cells by an immunomagnetic bead technique were cultured in NCS medium; the cells began to grow together and form cell spheres after 3–5 days. The cell spheres stained positive for nestin. Then the stem-like cell spheres were cultured by DMEM/F12 plus 10% FBS medium. Three days later, the stem-like cell spheres adhered to the well bottom and then thick dendrite-like pseudopodia grew from the spheres after one week. The differentiation cells stained positive for GFAP and *β*-tubulin (Figure 1).

**Figure 2:**
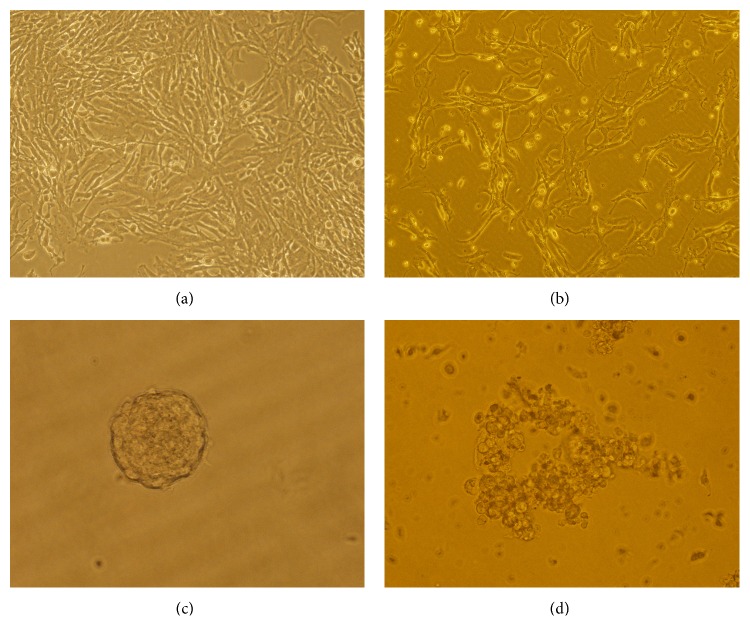
Cell morphology (10 × 20). (a) U251 cells cultured in normal medium. (b) U251 cells cultured in medium containing 100 *μ*g/mL intervention corilagin for 48 h. (c) U251 stem-like cells cultured in normal NSC medium. (d) U251 stem-like cells cultured in NSC medium containing 100 *μ*g/mL intervention corilagin for 48 h.

Under an inverted microscope, it was found that the U251 cells without corilagin intervention grew well with intact cell structure and were attached to the wall. With 100 *μ*g/mL corilagin intervention for 48 h, the cells became shrunken and cell density reduced with a little of cell fragments, and cell proliferation was inhibited. For the U251 stem-like cells, the cells grew well with sphere shape and were suspended in the medium without corilagin. After being treated by 100 *μ*g/mL corilagin intervention for 48 h, the quantity and density of cell spheres decreased, structure of cell spheres was destroyed with a great deal of cell debris, and cell proliferation was inhibited (Figure 2).

### 3.2. Effect of Corilagin on Cell Survival

**Figure 3:**
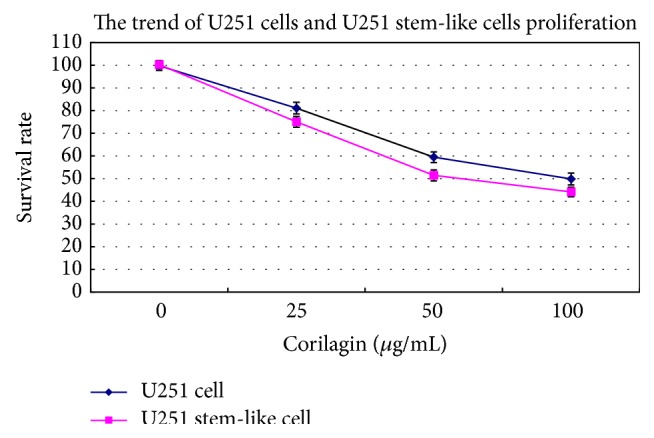
The proliferation trends of U251 cells and stem-like cells that were cultured treated with increasing concentrations of corilagin (0, 25, 50, and 100 *μ*g/mL) for 48 h.

The U251 cells and stem-like cells were treated with increasing concentrations of corilagin (0, 25, 50, and 100 *μ*g/mL) for 48 h; then the cell activity was detected using the CCK-8 assay. It was observed that the U251 cells proliferated faster than the U251 stem-like cells in the presence of the same corilagin concentration. Corilagin inhibited the cell proliferation of U251 stem-like cells and U251 cells, and the inhibition of U251 stem-like cells was stronger than that of U251 cells (*P* < 0.05, Figure 3).

### 3.3. Effect of Corilagin on Cell Cycle

**Figure 4:**
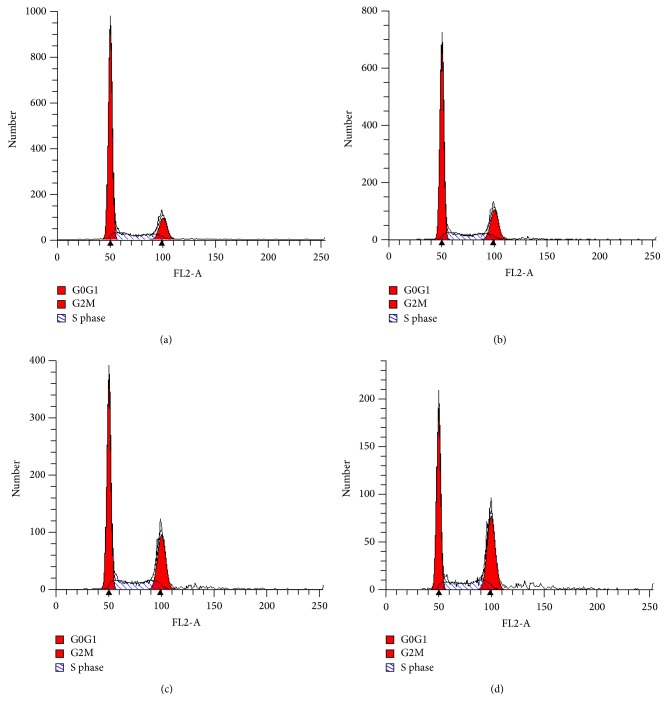
Cell cycle of U251 cells. (a) U251 cells cultured in normal medium; (b) U251 cells cultured in medium containing 25 *μ*g/mL intervention corilagin for 48 h; (c) U251 cells cultured in medium containing 50 *μ*g/mL intervention corilagin for 48 h; and (d) U251 cells cultured in medium containing 100 *μ*g/mL intervention corilagin for 48 h.

**Figure 5:**
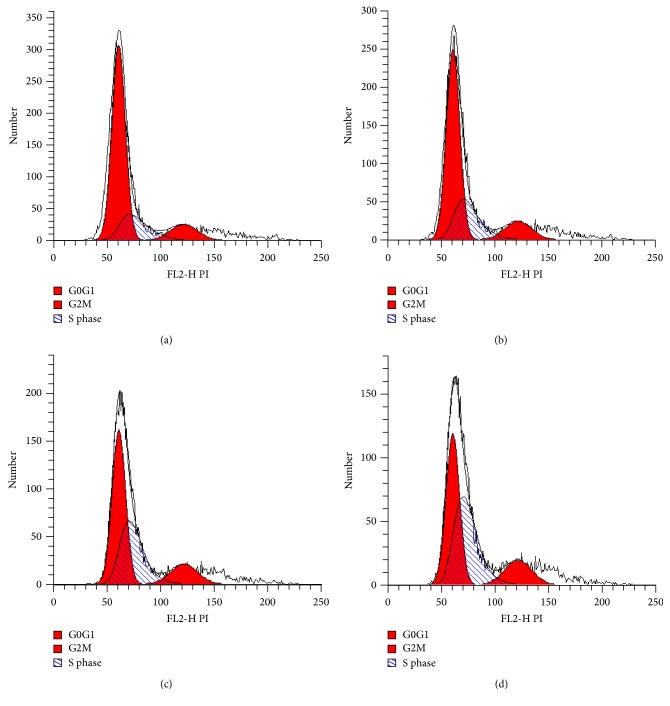
Cell cycle of U251 stem-like cells. (a) U251 stem-like cells cultured in normal medium; (b) U251 stem-like cells cultured in medium containing 25 *μ*g/mL intervention corilagin for 48 h; (c) U251 stem-like cells cultured in medium containing 50 *μ*g/mL intervention corilagin for 48 h; and (d) U251 stem-like cells cultured in medium containing 100 *μ*g/mL intervention corilagin for 48 h.

In the U251 cell group, after cells were treated by corilagin (0, 25, 50, and 100 *μ*g/mL) for 48 h, it had an increased frequency of cells in the G2/M phase. But, for the U251 stem-like cells, it had an increased frequency of cells in the S phase (*P* < 0.05; Table 1, Figures 4 and 5). These results indicate that the effect of corilagin on the cell cycle was different between U251 cells and U251 stem-like cells.

### 3.4. Effect of Corilagin on p65 Gene Promoter Expression

**Figure 6:**
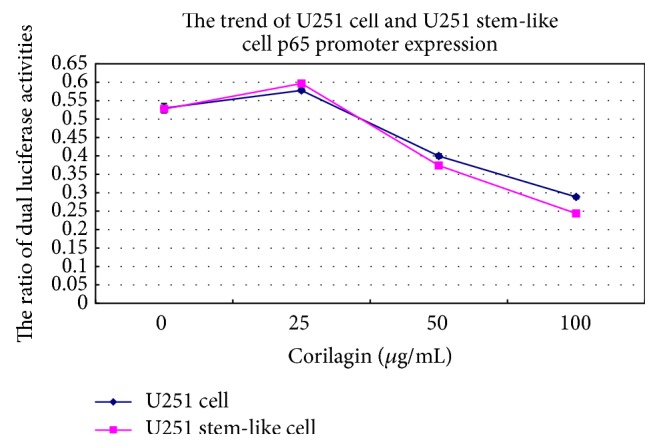
The p65 promoter expression trend of U251 cells and stem-like cells that were cultured treated with increasing concentrations of corilagin (0, 25, 50, and 100 *μ*g/mL) for 48 h.

Being treated with increasing concentrations of corilagin (0, 25, 50, and 100 *μ*g/mL) for 48 h, the U251 cells and stem-like cells were detected by dual luciferase assay. The results showed that the ratio of dual luciferase activities of U251 cells and stem-like cells increased at a low concentration of corilagin but decreased at higher corilagin concentration. The ratio of dual luciferase activities of U251 stem-like cells was lower compared to U251 cells' in the same concentration of corilagin. It indicated that corilagin inhibited the p65 gene promoter expression of U251 stem-like cells and U251 cells, and the inhibition of U251 stem-like cells was stronger than that of U251 cells (*P* < 0.05; Figure 6).

### 3.5. Effect of Corilagin on IKB*α* and p65 Protein Expression

**Figure 7:**
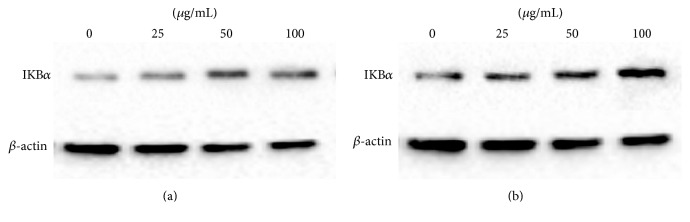
The IKB*α* protein expression trend of U251 cells and stem-like cells that were cultured treated with increasing concentrations of corilagin (0, 25, 50, and 100 *μ*g/mL) for 48 h. (a) U251 cell group. (b) U251 stem-like cell group.

**Figure 8:**
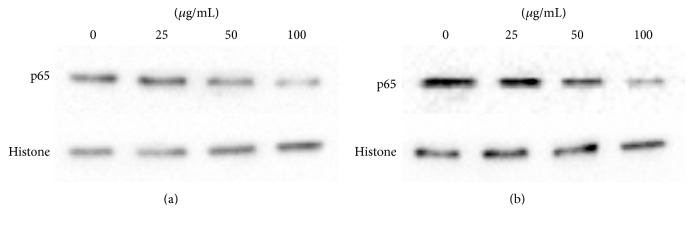
The p65 protein expression trend of U251 cells and stem-like cells that were cultured treated with increasing concentrations of corilagin (0, 25, 50, and 100 *μ*g/mL) for 48 h. (a) U251 cell group. (b) U251 stem-like cell group.

As a following result, with increasing concentrations of corilagin, the IKB*α* expression in cytoplasm of U251 cells and U251 stem-like cells was increased, but the p65 expression in nucleus of U251 cells and U251 stem-like cells was decreased (*P* < 0.05; Figures 7 and 8).

## 4. Discussion

GBM is the most common and malignant tumor in central nervous system whose growth is aggressive and was difficult to completely resect by surgery. Therefore, drug therapy plays an important role in eliminating remaining tumor cells in surgical treatment. With the in-depth research of antitumor effect of traditional Chinese medicine, there are more and more people paying close attention to the antitumor effects of Chinese herbal medicine at home and abroad. Studies had been reported that corilagin could inhibit the growth of SMMC7721 cells and Bel7402 cells and induce cell cycle arrest at the G2/M phase [11]. Corilagin could also inhibit the growth of Hep3B hepatoma cells, enhance the cytotoxicity of cisplatin and doxorubicin, and make a stronger destruction to tumor [12]. Moreover, it had no adverse effect on liver tissues [13].

Nuclear factor *κ*B (NF-*κ*B) is a nuclear transcription factor that regulates expression of a large number of genes that are critical for the regulation of apoptosis, viral replication, tumorigenesis, inflammation, and various autoimmune diseases. NF-*κ*B is widely expressed in human cells and composited by p65/p50 subunits dimer in most instances. In unstimulated cells, NF-*κ*B binds to the inhibitor protein, IKB*α*, and is sequestered in the cytoplasm. The various stimuli that activate NF-*κ*B cause phosphorylation and degradation of IKB*α*, and then the activated p65 translocates into the nucleus to regulate gene transcription. The nuclear NF kappa B1/p50 expression is dictated by its interaction with Inhibitor of NF-*κ*B*α* in astrocytomas and is associated with tumor grade and angiogenic factors [14]. NF-*κ*B is positively correlated with the malignant degree, the higher degree of malignancy, and the higher expression of NF-*κ*B. Lee et al. [15] revealed that high NF-*κ*B expression strongly correlates with rapid tumor progression and poor patient survival rates. Inhibiting the expression of NF-*κ*B could induce apoptosis of tumor [16].

Our present results denote that corilagin can inhibit the proliferation of U251 cells and U251 stem-like cells; the inhibitory effect on U251 stem-like cells was stronger than U251 cells in the presence of the same corilagin concentration. According to the results of cell cycle assay, we found that the U251 cells were arrested at G2/M phase (Mitosis) by corilagin and the U251 stem-like cells were arrested at S phase (DNA replication). The results of dual luciferase assay showed that a low concentration of corilagin could induce the expression of p65 gene promoter of U251 cells and U251 stem-like cells, while a higher concentration of corilagin could inhibit the expression. This effect was exerted more obviously on glioma stem-like cells than that on glioma cells. Our analysis is that corilagin can inhibit the proliferation of glioblastoma cells and glioblastoma stem-like cells, stagnating the cell cycle, inducing the expression of p65 gene promoter. With the increase of drug concentration, the inhibition on cell proliferation exceeds the inductive effect of p65 gene promoter expression, thereby inhibiting NF-*κ*B signaling pathway and promoting apoptosis of tumor cells. According to western blot test results, with increasing concentration of corilagin, the expression of IKB*α* protein in cytoplasm showed an increasing trend, while the expression of NF-*κ*B/p65 protein in nucleus showed a decreasing trend. In addition, the effect of corilagin on IKB*α* and p65 protein expression is more obvious in glioblastoma stem-like cells than that in glioblastoma cells. It shows that corilagin can induce the expression and inhibit the degradation of IKB*α*, block the activation of NF-*κ*B, reduce the activated p65 protein entering into the nucleus, thereby inhibit NF-*κ*B signaling pathway, and induce tumor cell apoptosis. These results confirm and expand the study of corilagin in NF-*κ*B signaling pathway area [17, 18].

In conclusion, we present evidence that corilagin can inhibit the proliferation of U251 cells and U251 stem-like cells; the inhibitory effect on U251 stem-like cells is stronger compared to U251 cells, which is contrary to the glioblastoma stem-like cells characteristic of antichemotherapy. This different result indicates that the effect of corilagin on U251 cells and U251 stem-like cells may have close relationships with mechanism of cell cycle and NF-*κ*B signaling pathway. Combining the research results and corilagin possible antitumor mechanism, we speculate that NF-*κ*B has a higher expression in glioblastoma stem-like cells, which make NF-*κ*B activity play a more important role in the proliferation of glioblastoma stem-like cells. Inhibiting the NF-*κ*B activity can make a more obvious apoptosis in glioblastoma stem-like cells than that in glioblastoma cells. In this study, corilagin, the active ingredient of Chinese herbal medicine, was applied to glioblastoma stem-like cell research, which could broaden the idea of glioblastoma treatment, providing a theoretical basis for the clinical treatment of malignant glioblastoma. However, this study still has some limitations; the real antitumor mechanism of corilagin is not yet clear and requires further study.

## Figures and Tables

**Table 1:** Effect of corilagin on cell cycle of U251 cells and U251 stem-like cells for 48 h (mean ± SD, %).

Groups	U251 cells			U251 stem-like cells		
	G0/G1	S	G2/M	G0/G1	S	G2/M
Control (0 *μ*g/mL)	69.03 ± 1.08	18.24 ± 0.33	12.73 ± 0.75	70.40 ± 1.16	17.91 ± 0.65	11.69 ± 0.94
25 *μ*g/mL	62.79 ± 0.45	19.40 ± 0.78	17.81 ± 0.91^*∗*^	62.27 ± 0.90	24.66 ± 0.68^*∗*^	13.07 ± 0.86
50 *μ*g/mL	52.31 ± 1.23	20.10 ± 0.25	27.59 ± 1.42^*∗*^	52.20 ± 0.90	34.48 ± 1.28^*∗*^	13.33 ± 0.49
100 *μ*g/mL	43.89 ± 1.04	21.27 ± 1.09	34.83 ± 1.29^*∗*^	42.07 ± 1.02	43.45 ± 1.27^*∗*^	14.48 ± 0.67

Note: *∗* represents *P* < 0.05 compared to normal control.
